# Possible Role of *Rickettsia felis* in Acute Febrile Illness among Children in Gabon

**DOI:** 10.3201/eid2110.141825

**Published:** 2015-10

**Authors:** Gaël Mourembou, Jean Bernard Lekana-Douki, Oleg Mediannikov, Sydney Maghendji Nzondo, Lady Charlene Kouna, Jean Claude Biteghe Bi Essone, Florence Fenollar, Didier Raoult

**Affiliations:** Ecole Doctorale Régionale d’Afrique Centrale, Franceville, Gabon (G. Mourembou);; Aix Marseille Université, Marseille, France (G. Mourembou, O. Mediannikov, F. Fenollar, D. Raoult);; Université des Sciences de la santé de Libreville, Libreville, Gabon (J.B. Lekana-Douki);; Centre International de Recherche Médicale de Franceville (CIRMF), Franceville (J.B. Lekana-Douki, S. Maghendji Nzondo, L.C. Kouna, J.C. Biteghe Bi Essone)

**Keywords:** Rickettsia felis, R. felis, bacteria, fever, febrile, unexplained fever, urban, rural, Gabon, sub-Saharan Africa, children, prevalence, acute febrile illness, rainy season, pathogenic bacteria, fastidious bacteria

## Abstract

*Rickettsia felis* has been reported to be a cause of fever in sub-Saharan Africa, but this association has been poorly evaluated in Gabon. We assessed the prevalence of this bacterium among children <15 years of age in 4 areas of Gabon; the locations were in urban, semiurban, and rural areas. DNA samples from 410 febrile children and 60 afebrile children were analyzed by quantitative PCR. Overall, the prevalence of *R. felis* among febrile and afebrile children was 10.2% (42/410 children) and 3.3% (2/60 children), respectively. Prevalence differed among febrile children living in areas that are urban (Franceville, 1.3% [1/77]), semiurban (Koulamoutou, 2.1% [3/141]), and rural (Lastourville, 11.2% [15/134]; Fougamou, 39.7% [23/58]). Furthermore, in a rural area (Fougamou), *R. felis* was significantly more prevalent in febrile (39.7% [23/58]) than afebrile children (5.0% [1/20]). Additional studies are needed to better understand the pathogenic role of *R. felis* in this part of the world.

## Abstract 2

Infection is widespread but most prevalent among young, rural residents with fever.

Over the past decade, reported cases of malaria and associated deaths have declined in Africa (*1*). This decrease has led to a search for other causes of fever in Africa, where unexplained febrile illnesses are one of the major health problems. In some sub-Saharan Africa countries, malaria treatments are still administered without a biologic diagnosis. For example, an assessment of complicated malaria and other severe febrile illness cases in a pediatric ward in Libreville, Gabon, showed that 43.5% of the children who received an antimalarial treatment had microscopy test results negative for malaria (*2*). 

Other studies have shown that, in addition to malaria, other bacterial infections are a major cause of fever in Africa (*3*–*6*). *Staphylococcus aureus*, *Streptococcus pneumoniae*, nontyphoidal *Salmonella* spp., *Klebsiella pneumoniae*, and *Escherichia coli* are the bacteria most often detected in sub-Saharan Africa by the culture method (*7*,*8*). The use of molecular tools has enabled the identification of the following fastidious bacteria as a cause of unexplained fevers in Africa: *Rickettsia* spp., including *R. felis* (*3*–*5*,*9*); *Coxiella burnetii* (*10*); *Tropheryma whipplei* (*11*); and *Borrelia* spp. (*12*,*13*). However, the epidemiology of many fastidious bacteria, such as *R. felis*, remains poorly understood. In rural areas of Senegal, the prevalence of *R. felis* was generally higher (7%–24%) than that in urban areas of sub-Saharan African, such as Franceville, Gabon (10%) (*14*). 

*R. felis* is a gram-negative bacterium belonging to the spotted fever group of *Rickettsia* spp. In Gabon, the bacterium has been reported in arthropods, including *Ctenocephalides felis* cat fleas (*15*) and *Aedes albopictus* mosquitoes (*16*), and in humans (*14*). Similar to many African countries, Gabon has a strong disparity between health care in urban and rural areas; in rural areas, little is known about the epidemiology of infectious diseases. The aim of our study was to evaluate the prevalence of *R. felis* infection among febrile and afebrile children in rural and urban areas of Gabon and the possible role of *R. felis* in acute febrile illness.

## Materials and Methods

### Study Area

**Figure 1:**
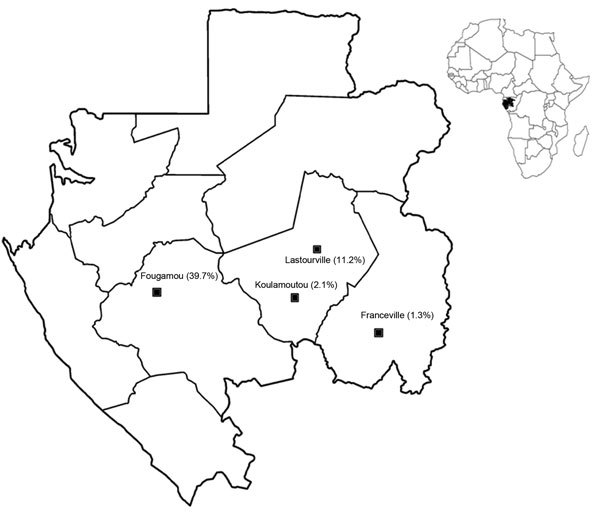
Four rural (Fougamou and Lastourville), semiurban (Koulamoutou), and urban (Franceville) locations in Gabon where children <15 years of age were tested for *Rickettsia felis* infection, April 2013–January 2014. Percentages in parentheses indicate the prevalence of *R. felis* infection among febrile children. Inset shows location of Gabon on the Atlantic Coast of Africa.

Gabon is a central African country located on the equator along the Atlantic Coast (Figure 1). The country has a low coastal plain and hilly inland areas and savannas to the east and south; 80% of Gabon is covered by forest. The tropical climate is hot and humid, and the seasons alternate in precipitation and length: short dry season, long rainy season, long dry season, short rainy season.

### Study Design and Participants

Patients were recruited at 4 health centers (Figure 1) located in 3 Gabon provinces. One center, the Regional Hospital Center Amissa Bongo of Franceville, is in an urban area of Haut-Ogooué Province. Two centers, the Regional Hospital Center Paul Moukambi of Koulamoutou and the Medical Center of Lastourville, are in semiurban and rural areas, respectively, of Ogooué Lolo Province. The fourth center, the Medical Research Unit of Ngounie in Fougamou, is in a rural area of Ngounié Province.

The National Ethics Committee of Gabon approved this prospective study (no. 0023/2013/SG/CNE). Written informed consent forms and questionnaires were completed by parents or legal guardians upon a child’s enrollment in the study.

During April 2013–January 2014, a total of 525 children <15 years of age were recruited for the study; 465 of the children were febrile (axillary temperature >37.5°C), and 60 were afebrile (controls). Febrile children were recruited from the pediatric outpatient clinics at the 4 health care centers. The control group was recruited from children who had accompanied their sick parents to the health care centers. Children in the control group had to be free of fever for at least 1 week before study inclusion. 

### Sample Collection and Molecular Analysis

Molecular analyses were performed on DNA extracts from blood samples from each child; blood smears, serologic testing, and culture were not done. After a child’s parent or legal guardian was interviewed, a blood sample was collected into an EDTA tube. World Health Organization guidelines for blood collection were followed, including guidelines for hand hygiene, use of sterile tubes, and skin disinfection with 70% alcohol. The International Center of Medical Research of Franceville, which has a well-trained staff with expertise in infectious diseases, performed DNA extraction by using the E.Z.N.A. Blood DNA Maxi Kit (Omega Bio-tek, Norcross, GA, USA) according to the manufacturer’s protocol (*17*). A total of 150 μL of DNA extract was obtained from each sample. The extracts were stored at −20°C before being sent on ice to URMITE (Unité de Recherche sur les Maladies Infectieuses et Tropicales, Marseille, France) for molecular analyses.

Specific quantitative PCR (qPCR) was performed by using a CFX96 Touch Real-Time PCR Detection System (Bio-Rad Laboratories, Marnes-la-Coquette, France). qPCR Master Mix (Eurogentec, Liege, Belgium) was prepared according to the manufacturer’s instructions; for each reaction, 15 μL of Master Mix was added to 5 μL of DNA. The quality of extracted DNA and the lack of PCR inhibitors were systematically checked by targeting a housekeeping gene, human β-actin (*14*). Positive (*R. felis* DNA) and negative (mix alone) controls were also systematically used for each PCR assay. All samples were screened by *Rickettsia* spp.–specific qPCR targeting the *gltA* gene and by *R. felis*–specific qPCR targeting the *bioB*, *orfB*, and *vapB1* genes (*14*) (Table 1). Samples positive for at least 2 genes were considered positive.

**Table 1:** Primers and probes used in *Rickettsia* spp.–specific *and R. felis*–specific quantitative qPCR screening of blood samples from children <15 years of age in Gabon*

Pathogens, target gene	Primers, forward; reverse 5'→3'	Probe, 5'→3'
*Rickettsia* spp		
RKND03 (*gltA*)	GTGAATGAAAGATTACACTATTTAT GTATCTTAGCAATCATTCTAATAGC	6FAM-CTATTATGCTTGCGGCTGTCGGTTC-TAMRA
*R. felis*		
*bioB* (*0527*)	ATGTTCGGGCTTCCGGTATG CCGATTCAGCAGGTTCTTCAA	6FAM-GCTGCGGCGGTATTTTAGGAATGGG-TAMRA
* orfB *	CCCTTTTCGTAACGCTTTGCT GGGCTAAACCAGGGAAACCT	6FAM-TGTTCCGGTTTTAACGGCAGATACCCA-TAMRA
* vapB1 *	TGTCTTTCATGAATTGATCAGCA AGGCGAAAGCTTTGACGTG	6FAM-AAGGCTTGGTTTCTGCGGGC-TAMRA

*The origin of the quantitative PCR is described in (*14*).

### Statistical Analysis

Data were analyzed by using Epi Info software version 7.0.8.0 (Centers for Disease Control and Prevention, Atlanta, GA, USA). Mantel-Haenszel χ^2^ and Fisher exact tests were used to compare the prevalence between groups (i.e., febrile and afebrile children), seasons, and geographic areas and by the participants’ sex and age. A 2-tailed p value <0.05 was considered statistically significant.

## Results

### Recruitment

A total of 465 febrile children were recruited from Franceville (n = 80), Koulamoutou (n = 167), Lastourville (n = 155), and Fougamou (n = 63) (Table 2). However, 55 of these children were excluded from the statistical analysis because their sex and age data were unavailable; 3 of the excluded children were from Franceville, 26 from Koulamoutou, 21 from Lastourville, and 2 from Fougamou. Of the 410 children included in the statistical analysis, 52.7% (216/410) were recruited during a rainy season, and 47.3% (194/410) were recruited during a dry season. A total of 60 afebrile children were recruited and enrolled in parallel from Franceville (n = 24), Fougamou (n = 20), and Lastourville (n = 16); no afebrile children were enrolled from Koulamoutou. The 410 febrile patients included in the statistical analysis consisted of 212 boys and 198 girls (sex ratio 1.07). 

**Table 2:** *Rickettsia felis* test results and demographic data for children recruited for sampling in Gabon, April 2013–January 2014*

Site	Population	No. *R. felis* positive/no. tested (%)		Latitude, longitude	Altitude, m	Lifestyle	Vegetation
		Febrile children	Afebrile children				
Franceville	≈56,000	1/77 (1.3)	1/24 (4.2)	1°37′14.52′′S, 13°34′57.72′′E	333	Urban	Savannah
Koulamoutou	≈17,000	3/141 (2.1)	None enrolled	1°8′20.65′′S, 12°28′0.2′′E	349	Semiurban	Plantations and degraded forest
Lastourville	≈10,000	15/134 (11.2)	0/16	0°49′1.2′′S, 12°42′0′′E	483	Rural	Rainforest
Fougamou	≈4,100	23/58 (39.7)	1/20 (5.0)	1°13′0.01′′S, 10°36′0′′E	108	Rural	Plantations and degraded forest

*For all sites, the short dry season occurred during mid-December–mid-February, the short rainy season occurred during mid-February–mid-May, the long dry season occurred during mid-May–mid-September, and the long rainy season occurred during mid-September to mid-December.

### *R. felis* in Febrile and Afebrile Children in Gabon

**Figure 2:**
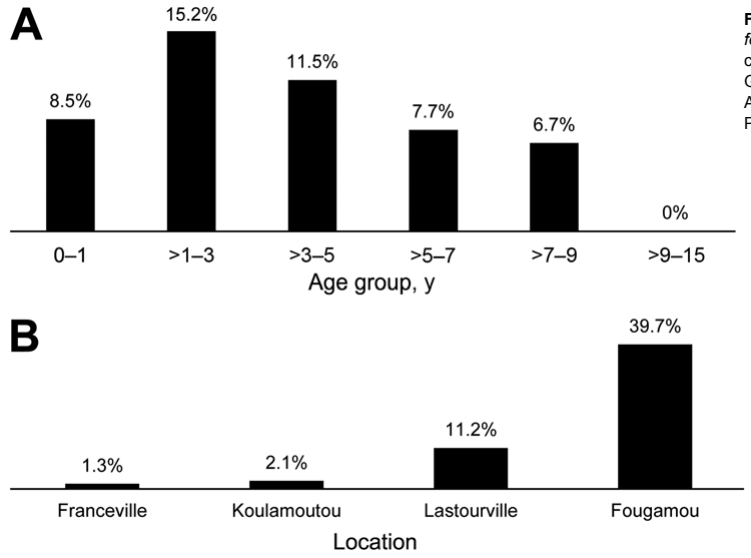
Prevalence of *Rickettsia felis* infection among febrile children <15 years of age in Gabon, April 2013–January 2014. A) Prevalence by age group. B) Prevalence by location.

*R. felis* DNA was detected in 42 (10.2%) of 410 analyzed samples from febrile children (Table 3). The bacterium was detected significantly more frequently during the rainy season (15.3% [33/216 samples]) than the dry season (4.6% [9/194 samples]; p<0.001). The prevalence among boys (10.8% [23/212]) and girls (9.6% [19/198]) did not differ significantly (p = 0.74). Among febrile children, *R. felis* prevalence varied by age group: 8.5% (11/129 children) among children 0–1 year of age, 15.2% (16/105) among children >1– 3 years of age, 11.5% (10/87) children >3–5 years of age, 7.7% (3/39) among children >5–7 years of age, 6.7% (2/30) among children >7–9 years of age, and 0 (0/20) among children >9–15 years of age (Figure 2, panel A). The prevalence of *R. felis* among febrile children did not differ substantially by age, but the prevalence did increase progressively from Franceville (1.3% [1/77]) to Koulamoutou (2.1% [3/141]) to Lastourville (11.2% [15/134]) to Fougamou (39.7% [23/58]); however, no adjustments were made when testing these pairs (Figure 2, panel B). The prevalence was statistically lower in Franceville than in Lastourville (odds ratio [OR] 0.1, 95% CI 2.5 × 10^−3^–0.7; p = 0.006), in Franceville than in Fougamou (OR 0.02, 95% CI 5 × 10^−4^–0.1, p<0.001), in Koulamoutou than in Lastourville (OR 0.17, 95% CI 0.03–0.63; p = 0.002), in Koulamoutou than in Fougamou (OR 0.03, 95% CI 6 × 10^−3^–0.12; p<0.001), and in Lastourville than in Fougamou (OR 0.19, 95% CI 0.08–0.43; p<0.001). Overall, the prevalence of *R. felis* among febrile children was significantly higher in the rural areas (Lastourville and Fougamou; 19.8% [38/192 children]) than in the urban area (Franceville; 1.3% [1/77 children]; p<0.001). Among the 60 afebrile children, only 2 (3.3%; both girls) were positive for *R. felis*; the girls were 1 and 3 years of age and were from Fougamou and Franceville, respectively. In all, *R. felis* DNA was detected in 10.2% (42/410) of febrile children and in 3.3% (2/60) of afebrile children; this difference was not significant (p = 0.09).

**Table 3:** Prevalence of *Rickettsia felis* infection among febrile and afebrile children <15 years of age, Gabon, April 2013–January 2014*

Location, participants’ fever status	No. positive children/no. tested (%), by age, y							
	0–1	>1–3	>3–5	>5–7	>7–9	>9–15		All ages
Fougamou								
Febrile	7/22 (31.8)	8/11 (72.7)	4/11 (36.4)	2/6 (33.3)	2/5 (40.0)	0/3		23/58 (39.7)
Afebrile	1/5 (20.0)	0/4	0/3	0/3	0/2	0/3		1/20 (5.0)
Lastourville								
Febrile	4/38 (10.5)	7/42 (16.7)	3/34 (8.8)	1/9 (11.1)	0/8	0/3		15/134 (11.2)
Afebrile	0/2	0/6	0/5	0/1	0/2	NA		0/16
Koulamoutou†								
Febrile	0/55	0/35	3/24 (12.5)	0/14	0/7	0/6		3/141 (2.1)
Franceville								
Febrile	0/14	1 /17 (5.8)	0/18	0/10	0/10	0/8		1/77 (1.3)
Afebrile	0/10	1/7 (14.2)	0/1	0/4	0/2	NA		1/24 (4.2)
Total cases	12/146 (8.2)	17/122 (13.9)	10/96 (10.4)	3/47 (4.1)	2/36 (5.5)	0/23		44/470 (9.4)

*NA, not available. †No afebrile children in Koulamoutou were enrolled in the study.

### *R. felis* at Each Sampling Location

#### Fougamou, Ngounié Province

*R. felis* DNA was detected in 23 (39.7%) of 58 febrile children at this rural location (Table 3; Figure 2). Prevalence during the rainy season (48.7% [19/39 children]) was higher than that during the dry season (21.1% [4/19 children]; p = 0.05). *R. felis* prevalence among boys (50% [15/30]) and girls (28.6% [8/28]) did not differ significantly (p = 0.11). *R. felis* prevalence also did not differ significantly by age, even though prevalence was higher among 1- to 3-year-old children (Table 3). Overall, *R. felis* DNA was detected significantly more frequently in febrile (39.7% [23/58]) than afebrile (5.0% [1/20]) children (p = 0.004).

#### Lastourville, Ogooué Lolo Province

*R. felis* DNA was detected in 15 (11.2%) of 134 febrile children in this rural location (Table 3; Figure 2). *R. felis* prevalence during the rainy season (15.8% [12/76 children]) was higher than that during the dry season (5.2% [3/58 children]), but the difference was not statistically significant (p = 0.05). Prevalence among boys (7.6% [6/79]) and girls (16.4% [9/55]) did not differ significantly (p = 0.17). Prevalence also did not differ significantly by age (Table 3). *R. felis* DNA was detected in 15 (11.2%) of 134 febrile children and in 0 of 16 afebrile children; this difference was not statistically significant (p = 0.3).

#### Koulamoutou, Ogooué Lolo Province

*R. felis* DNA was detected in 3 (2.1%) of 141 febrile children in this semiurban location (Table 3; Figure 2). Prevalence during the rainy season (2.9% [2/68 children]) was higher than that during the dry season (1.4 [1/73 children]; p = 0.6). *R. felis* DNA was detected in 1 (1.6%) of 63 boys and in 2 (2.6%) of 78 girls (p<1); 2 of these children were 4 years of age, and 1 was 5 years of age. As stated above, no afebrile children were enrolled from Koulamoutou.

#### Franceville, Haut-Ogooué Province

*R. felis* DNA was detected in 1 (1.3%) of 77 febrile children and in 1 (4.2%) of 24 afebrile children (p = 0.3) in this urban area (Table 3; Figure 2). The infected febrile child was a 2-year-old boy, and the infected afebrile child was a 3-year-old girl; both children became infected during the dry season. 

## Discussion

The lack of molecular tools in many health centers in countries in sub-Saharan Africa limits the management of all febrile illnesses in these areas. In this study, we used molecular tools (PCR assays) to assess the prevalence of *R. felis* in blood specimens from febrile and afebrile children from rural, semiurban, and urban areas of Gabon. One of the most frequent pitfalls of PCR assays is the contamination of samples, which can occur any time during or after collection of the samples, including during their use in the laboratory. Over the years, the URMITE laboratory has developed strategies and applied rigorous procedures to prevent and detect contamination (*18*–*21*). For example, we require that 2 different PCR assays show positive results before we conclude that a sample is positive (*21*).

 In this study, we used a *Rickettsia* spp.–specific qPCR targeting the highly conservative *gltA* gene and an *R. felis*–specific qPCR targeting the *bioB*, *orfB*, and v*apB1* genes. The *Rickettsia* spp.–specific assay can amplify almost all *Rickettsia* spp., including *R. conorii* and *R. rickettsii*, but it is less sensitive than the *R. felis*–specific assay. When a sample in our study was positive by the *Rickettsia* spp.–specific qPCR, it was also positive by the *R. felis* qPCR. Several samples had positive results for only 2 of the 3 genes targeted by the *R. felis*–specific qPCR. This discrepancy might be explained by possible genetic diversity of the *R. felis* strains. The 3 genes used for the *R. felis*–specific assay are not all conservative, so it is possible that some strains have a certain degree of genetic diversity that may occasionally cause false-negative PCR results. Our results were also systematically validated by using rigorous criteria. To check the quality of each PCR run, we used negative controls (i.e., PCR mix without template) with every tenth sample, and we used 2 positive controls (*R. felis* DNA) per run. In addition, we required that all negative and positive controls be systematically correct (i.e., negative and positive, respectively) before validating each PCR run, and a sample was not considered *R. felis*–positive unless confirmed by at least 2 of the 4 sequences of targeted DNA. Thus, we consider our results to be valid. The fact that *R. felis* DNA has not been detected in samples (from febrile patients in France and Tunisia) previously analyzed in our laboratory (*14*) provides further support of our team’s ability to prevent and detect contamination during PCR runs. Furthermore, it has been reported that the prevalence of *R. felis* is low in northern Africa countries (France, Algeria, Morocco, Tunisia) and increases in southern Africa countries (Mali, Senegal, Gabon) (*14*).

Consistent with our previous findings from Franceville in Haut-Ogooué Province (*14*), our findings from this study confirmed the presence of *R. felis* bacteremia in febrile children in Gabon and showed that the prevalence of infection was higher in rural than urban and semiurban areas. This fastidious bacterium was previously found in arthropods in Franceville (*15*), including the cat flea, *C. felis*. The bacterium was also detected in *A. albopictus* mosquitoes in Libreville in Estuaire Province (*16*). Data from our study also confirm the presence of *R. felis* in children in Ogooué lolo and Ngounié Provinces. Therefore, *R. felis* is widespread in Gabon, and its prevalence should be assessed in other areas of the country.

Common microorganisms involved in bacteremia (*S. aureus*, *Streptococcus pyogenes*, *E. coli*, *K. pneumoniae*, *Salmonella* spp., and *S. pneumoniae*) were previously assessed in Gabon by using standard culture methods (*8*), but the prevalence of fastidious bacteria, which are mainly detected by using molecular techniques, was not studied. There is a need to include these sensitive methods in diagnostic determinations. Our findings, plus those from studies in Senegal (*14*,*22*), Mali (*14*), Kenya (*5*), Ethiopia (*23**,**24*), Cameroon (*25*), Democratic Republic of the Congo (*26**,**27*), Ivory Coast (Côte d’Ivoire) (*6*), and Zimbabwe (*28*), show that *R. felis* is widespread in sub-Saharan Africa countries (*29*). However, its prevalence changes according to the season, year, area (rural, urban, and semiurban), country, and age of those infected.

A comparison of our findings with previously reported data showed that *R. felis* prevalence among febrile children in Franceville decreased from 10% in 2012 (*14*) to 1.3% in 2014. In addition, the variation in the *R. felis* prevalence between urban (1.3%) and rural (39.7%) areas of Gabon showed that *R. felis* is unequally distributed in the country. Differences in the prevalence of a possible vector or reservoir, or both, and disparate health care–associated conditions, including environmental conditions, poverty, and the availability and quality of health care facilities, between rural and urban areas may influence the distribution of *R. felis* in Gabon; however, these factors have not been determined. An increased prevalence of *R. felis* was observed during the rainy season. This same finding was described in Senegal, where the prevalence of infection in the rural areas was 24 times higher than that in urban areas of Algeria (*14*). Together, the finding suggests that *R. felis* is more prevalent in rural areas and during the rainy season in sub-Saharan Africa.

*R. felis* was previously found in *C. felis* fleas from a pet monkey in Gabon, but the data concerned only 1 region, Franceville (*15*). *R. felis* has been reported to be absent from *C. felis* fleas in rural areas of Senegal, where *R. felis* is common (*30*). The factors explaining the spread of *R. felis* in Gabon should be evaluated in further studies. Although *R. felis* is widespread in Africa and unequally distributed in Gabon and Senegal (*14*), its prevalence varies by country: 15.0% in Senegal (*14*), 3.0% in Mali (*14*), and 7.2% in Kenya (*5*). In our study, *R. felis* was mainly detected in young febrile children <5 years of age (primarily in those 1–3 years of age). In a rural area of Senegal (Dielmo and Ndiop), the incidence of *R. felis* infection has also been reported to be higher (reaching 36%) among febrile children 1–3 years of age, but the incidence was lower (0.1%) in persons >15 years of age (*14*). It has been reported that the seroprevalence of *R. felis* increases with age in areas where the bacterium is endemic (*5*). This increase is probably due to exposure to the bacterium during the course of a lifetime, leading to protection by a progressive development of immunity against this bacterium in adults.

Most of the fever-associated studies conducted in Africa failed to use a control group of afebrile persons. Consequently, when a pathogen was detected in febrile patients, it was systematically and automatically considered as the cause of fever (*7*). In some cases, we have also observed a mistake in methodology: data comparisons were performed between samples from afebrile persons in an occidental area and from febrile persons in Africa (*31*). The epidemiology of microorganisms depends on the studied areas, and the positive predictive value of a disease depends on its symptoms and epidemiology. For example, *Plasmodium falciparum*, the primary agent of malaria, is commonly detected in blood specimens from apparently healthy, afebrile persons in Sub-Saharan Africa; prevalence can reach 20% in Ethiopia and 32% in Senegal (*32**,**33*). Respiratory viruses, including influenza virus, have also been found in 12% of nasopharyngeal samples of asymptomatic Hajj pilgrims (*34*). More recently in Tanzania, the prevalence of *S. pneumoniae* DNA was less frequently detected in febrile (5.1%) than afebrile (6.3%) persons (*35*). Thus, these examples show that even well-known pathogens may be detected in blood or respiratory secretions of afebrile persons. The inclusion of control groups of participants in studies is indispensable to a better understanding of infectious diseases; the use of controls has shown the existence of carriers of well-known pathogens, emphasizing that it is not easy to interpret data about the potential pathogenic role of a microorganism. The finding of *R. felis* in febrile versus afebrile persons is not well characterized. The overall prevalence of *R. felis* in Gabon was higher in febrile (10.2%) than afebrile (3.3%) children, but the difference was not statistically significant (p = 0.09). Of more interest, in rural Fougamou, the difference in prevalence was significantly higher in similarly aged children with and without fever (39.7% vs. 5.0%, respectively; p = 0.004). Therefore, the presence of *R. felis* in febrile and afebrile persons should not exclude that this bacterium is a cause of fever in sub-Saharan Africa.

In 2010, independent research teams detected *R. felis* in blood specimens from febrile patients in 2 different areas of Africa (eastern and western) (*3*,*4*). In other studies, these teams confirmed and extended the preliminary data: 1 team showed that the presence of *R. felis* was 2.2 times higher in blood specimens from febrile persons compared with afebrile persons in Kenya (*5*), and the other team showed that the prevalence of *R. felis* was significantly higher in febrile (15.0%) than afebrile (4.0%) persons in Senegal (*14*). In Senegal, an 8-month-old febrile girl was cured of *R. felis* infection after treatment with doxycycline (*36*). The presence of *R. felis* has also been observed in blood specimens from febrile patients in Asia (*37*). The higher prevalence of *R. felis* among febrile persons compared with healthy persons in our study led us to suspect that this microorganism plays the role of pathogen. However, the presence of the microorganism may be a cofactor or the cause of a previous event not yet determined. Another hypothesis would be that blood specimens may be contaminated by surface bacteria, including *R. felis*, which has been detected on the skin of healthy persons in Senegal (*38*).

In summary, the *R. felis* bacterium is widespread in Gabon, but it primarily occurs in rural areas and is most prominent during the rainy season. *R. felis* is also more prevalent among febrile than afebrile children in rural areas of Gabon. More studies will help to better understand the pathogenic role of *R. felis* in this part of the world.
